# Supplementary figures and images for: Synthesis of Polyethylene Terephthalate (PET) with High Crystallization and Mechanical Properties via Functionalized Graphene Oxide as Nucleation Agent
Source: Molecules. 2024 Apr 24;29(9):1953. doi: 10.3390/molecules29091953 (PMC11085443; doi:10.3390/molecules29091953)

Appendix A. Supplementary data

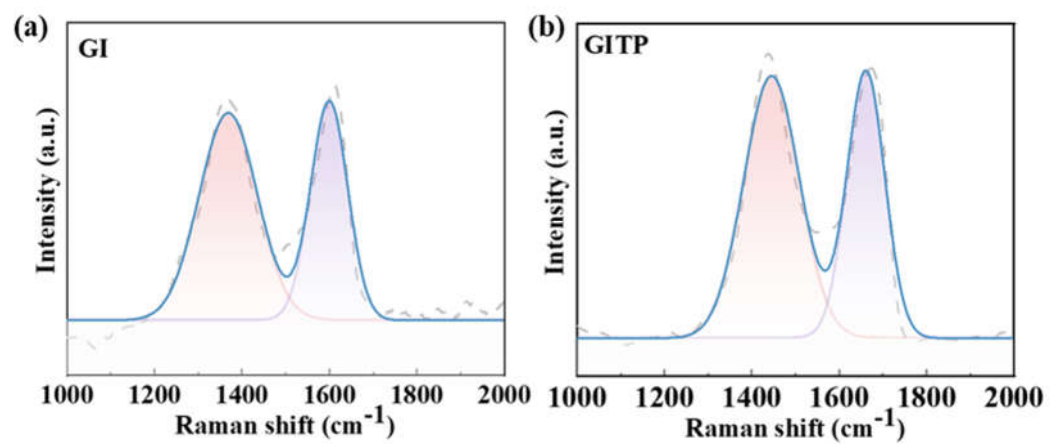

Figure S1. Raman fit plots for GI and GTP

Supplement: Supplementary file 1 [file molecules-29-01953-s001.zip › molecules-2947790-supplementary.pdf]
